# Complete Genome Sequence of *Enterobacter* sp. Strain ODB01, a Bacterium That Degrades Crude Oil

**DOI:** 10.1128/genomeA.01763-16

**Published:** 2017-03-09

**Authors:** Hui Lan, Hui Yang, Peiwang Li, Chong Wang, Haiyan Zhou, Hui Zhou, Hu Pan, Ye Yu, Xiangyang Lu, Yun Tian

**Affiliations:** a Hunan Province University Key Laboratory for Agricultural Biochemistry and Biotransformation, Hunan Agricultural University, Changsha, China; b College of Bioscience and Biotechnology, Hunan Agricultural University, Changsha, China; c Hunan Academy of Forestry, Changsha, China; d Hunan Co-Innovation Center for Utilization of Botanical Functional Ingredients, Changsha, China; e Institute of Agricultural Product Quality Standard and Testing Research, Tibet Academy of Agricultural and Animal Husbandry Sciences, Lhasa, China

## Abstract

*Enterobacter* sp. strain ODB01, which was isolated from the Changqing oil field, can degrade crude oil efficiently and use crude oil as its sole source of carbon and energy. We report the complete genome sequence of ODB01. The results promote its application in the remediation of petroleum contaminants.

## GENOME ANNOUNCEMENT

Crude oil, also known as petroleum, is unrefined oil exploited from oilfields and contains large amounts of complex hydrocarbons such as paraffins, aromatics, cycloalkanes, and alkenes. Toxic chemicals in the crude oil emphasize the importance of isolating beneficial microorganisms in the bioremediation of oil spills and deep-sea drilling sites (1). Numerous strains, which commonly belong to *Pseudomonas* sp., *Acinetobacter* sp., *Bacillus* sp., and *Dietzia* sp., were screened and categorized based on their oil degradation abilities (2). Previous studies have demonstrated monooxygenase and/or dioxygenase as rate-limited enzymes that control petroleum degradation (3). Therefore, it is particularly important to mine crude oil-degrading microorganisms with various oxygenase activities when performing the bioremediation of the crude oil contaminants. The *Enterobacter* sp. strain ODB01, which was isolated from the Changqing oilfield, can degrade crude oil efficiently and use crude oil as its sole source of carbon and energy. Strain ODB01 is a Gram-negative, aerobic, motile, and rod-shaped strain, but little has been known about the molecular mechanism of strain ODB01 in crude oil degradation, so we sequenced the complete genome of *Enterobacter* sp. strain ODB01.

The genomic DNA of ODB01 was extracted using the TaKaRa MiniBEST bacteria genomic DNA extraction kit. The quantity and quality of genomic DNA were assessed on the Agilent 2100 Bioanalyzer (Agilent, USA). Genomic DNA of strain ODB01 was sequenced using single molecular sequencing technology with the PacBio RS II system (Nextomics Biosciences, Wuhan, China) (4). *De novo* assembly was accomplished using the PacBio hierarchical genome assembly process (HGAP2.2.3) (5). The gene sequence was predicted using GLIMMER (Gene Locator and Interpolated Markov ModelER) software (version 3.02), which adopts the interpolated Markov models (IMMs) (6). The clustered regularly interspaced short palindromic repeats (CRISPRs) were identified using CRISPRs Finder (7).

This workflow resulted in one contig, a circular chromosome with 4,534,036 bases in the ODB01 strain. This chromosome consists of 54.81% G+C content, 4,117 predicted coding sequences (CDSs), nine rRNA (5S) genes, eight rRNA (16S) genes, eight rRNA (23S) genes, 88 tRNA genes, eight noncoding (ncRNAs), and 61 pseudogenes. In addition, four CRISPRs clusters were identified by CRISPRs Finder, which might provide acquired resistance against foreign genetic material (8).

The analysis of the *Enterobacter* sp. strain ODB01 annotated genome prompted the discovery of multiple oxygenase genes that may act as key enzymes for hydrocarbon-pollutant degradation pathways, including quercetin 2,3-dioxygenase, anthranilate 1,2- dioxygenase, 3,4-dihydroxyphenylacetate 2,3-dioxygenase, vanillate O-demethylase oxygenase, naphthalene 1,2-dioxygenase, quinol monooxygenase, taurine dioxygenase, acireductone dioxygenase, alkanesulfonate monooxygenase, toluene-4-sulfonate monooxygenase, pyrimidine monooxygenase, monooxygenase MoxC, dioxygenase AlkB, and other monooxygenases. Based on this knowledge, aromatics are believed to be bioconverted to salicylic acid through the actions of a series of oxygenases, hydroxylases, reductases, and decarboxylase, whereas alkanes can be oxidized by monooxygenases such as MoxC in strain ODB01.

The above results provide preliminary insight into the hydrocarbon degradation in *Enterobacter* sp. strain ODB01. Further research on the genome information of strain ODB01 can clarify the mechanism required for crude oil degradation and highlight strain ODB01 as a promising prospect in the bioremediation of crude-oil contaminants.

### Accession number(s).

The complete genome sequence of *Enterobacter* sp. strain ODB01 was deposited in GenBank under accession no. CP015227.
